# Major adverse cardiovascular events associated with testosterone treatment: a pharmacovigilance study of the FAERS database

**DOI:** 10.3389/fphar.2023.1182113

**Published:** 2023-07-12

**Authors:** Hui Zhao, Jun-Min Li, Zi-Ran Li, Qian Zhang, Ming-Kang Zhong, Ming-Ming Yan, Xiao-Yan Qiu

**Affiliations:** ^1^ Department of Pharmacy, Huashan Hospital, Fudan University, Shanghai, China; ^2^ School of Pharmacy, Fudan University, Shanghai, China

**Keywords:** pharmacovigilance, testosterone treatment, TT, major adverse cardiovascular events, MACE, FAERS

## Abstract

**Background and purpose:** Testosterone is an essential sex hormone in maintaining masculine characteristics, which is prescribed for male hypogonadism as testosterone replacement treatment (TRT). Herein, we investigated long-standing controversies about the association between TRT and major adverse cardiovascular events (MACEs), based on real world adverse event (AE) reports, registered in the Food and Drug Administration Adverse Event Reporting System (FAERS).

**Methods:** Publicly available FAERS data from 1 January 2004 to 31 December 2022 were retrieved from the Food and Drug Administration (FDA) website. The data mining protocol including the reporting odds ratio (ROR) and the Bayesian confidence propagation neural network (BCPNN) was applied to analyze overreporting caused by risk factors and MACEs, including TRT, morbidities, and ages. The ROR and the BCPNN were also applied to investigate the annually developing trend of pharmacovigilance (PV) signals in the real world, retrospectively.

**Results:** A total of 3,057 cases referring to MACEs, with a median age of 57 years old (yo), were identified from 28,921 cases of testosterone users. MACEs related to PV signals have emerged since 2014, including cardiac death, non-fatal myocardial infarction, and non-fatal stroke. Myocardial infarction (MI) (ROR: 9.46; IC_025_: 3.08), acute myocardial infarction (AMI) (ROR: 16.20; IC_025_: 3.72), ischemic cardiomyopathy (ROR: 11.63; IC_025_: 2.20), and cardiomyopathy (ROR: 5.98; IC_025_: 1.96) were the most significant signals generated, and weaker signals included cardiac failure acute (ROR: 4.01; IC_025_: 0.71), cardiac arrest (ROR: 1.88; IC_025_: 0.56), and ventricular fibrillation (VF) (ROR: 2.38; IC_025_: 0.38). The time-to-onset (TTO) of MACEs was calculated with a median of 246 days for AMI.

**Conclusion:** For myocardial infarction and cardiomyopathy, TRT statistically tended to increase the risk of MACEs, while for cardiac arrhythmia, cardiac failure, and stroke, TRT demonstrated beneficial effects among the population with morbidities, such as testosterone deficiency (TD), diabetes mellitus (DM), and hypertension. MACEs were rare but led to serious outcomes including significant increase in death and disability. Since 2018, and before 2014, reports referring to TRT associated with MACEs were relatively scarce, which indicated that there might be a considerable number of cases that went unrecorded, due to neglection. Health workers and testosterone users might pay more attention to testosterone-induced MACEs.

## 1 Introduction

Testosterone is essential for the maintenance of muscle mass, sex drive, bone density, and fertility of men. Testosterone deficiency (TD) could be caused by various conditions, and testosterone is widely used for testosterone replacement therapy (TRT), to restore the testosterone level of men with hypogonadism or with low testosterone levels (low-T), which is considered as a safe and effective hormone supplement (Elliott et al., 2017; Bhasin et al., 2018) and treatment for certain types of cancers, such as hormone therapy for prostate cancer (Bhasin et al., 2018).

However, the necessity of testosterone treatment (TT), especially the association between TRT and major adverse cardiovascular events (MACEs) remains controversial. MACEs refer to negative outcomes related to the heart and blood vessels, including cardiac death, non-fatal infarction, and non-fatal stroke**,** which is a significant concern in the management of various medical conditions, including heart diseases and diabetes (Casas et al., 2021; Rini et al., 2022). Although there are a few available guidelines up till date, no conclusions were given on the association between TT and MACE risk (Minhas et al., 2021; Isidori et al., 2022). Various reviewed experimental studies on androgen administration in animal models concluded that androgen exposure increases the cardiotoxicity of androgens via mROS generation and NLRP3 inflammasome activation, while it also demonstrates cardioprotective effects of resistance training on the heart tissue by increasing the level of malondialdehyde (MDA) and protein carbonyl and reducing the risks of heart injuries and other issues affecting the heart including hypertrophy, fibrosis, autonomic imbalance, and the irreversible destruction of the heart tissue (Sessa et al., 2022).

Skeptical opinions were publicized claiming that TT might induce sudden cardiac death (Esposito et al., 2023), cardiomyopathy (Doleeb et al., 2019), and venous thrombosis (Bertola et al., 2017; Poirier-Blanchette et al., 2021), increasing the risk of myocardial infarction (MI), stroke (Vigen et al., 2013), and non-fatal myocardial infarction (Finkle et al., 2014). In 2014, the Food and Drug Administration (FDA) Advisory Committee agreed that enough attention should be paid to the potential testosterone-induced cardiovascular (CV) risks (Garnick, 2015; Seftel, 2015). In 2022, Bhasin et al. (2022) excised a study including 6,000 subjects and determined that TT in middle-aged and older men with hypogonadism was with or at increased risk of CV diseases. On the contrary, there were other publications that pointed out that TT has no effect on MACEs or that it even presented some beneficial effects (Wang et al., 2011; Baillargeon et al., 2015; Cheetham et al., 2017), including statements that TT is associated with a decrease in atherosclerosis, hypertension, intima–media thickness of carotid arteries, insulin resistance, and mortality in men due to all causes (Morgentaler et al., 2015) and no short-term increased risk of adverse events (AEs) is observed among subjects with hypogonadism (Elliott et al., 2017).

Herein, based on the FDA Adverse Event Reporting System (FAERS), we aim to solve long-standing controversies about the association between TRT and MACEs, using the reporting odds ratio (ROR) method in tandem with the Bayesian confidence propagation neural network (BCPNN).

## 2 Methods

### 2.1 Data source and the scheme

**FIGURE 1:**
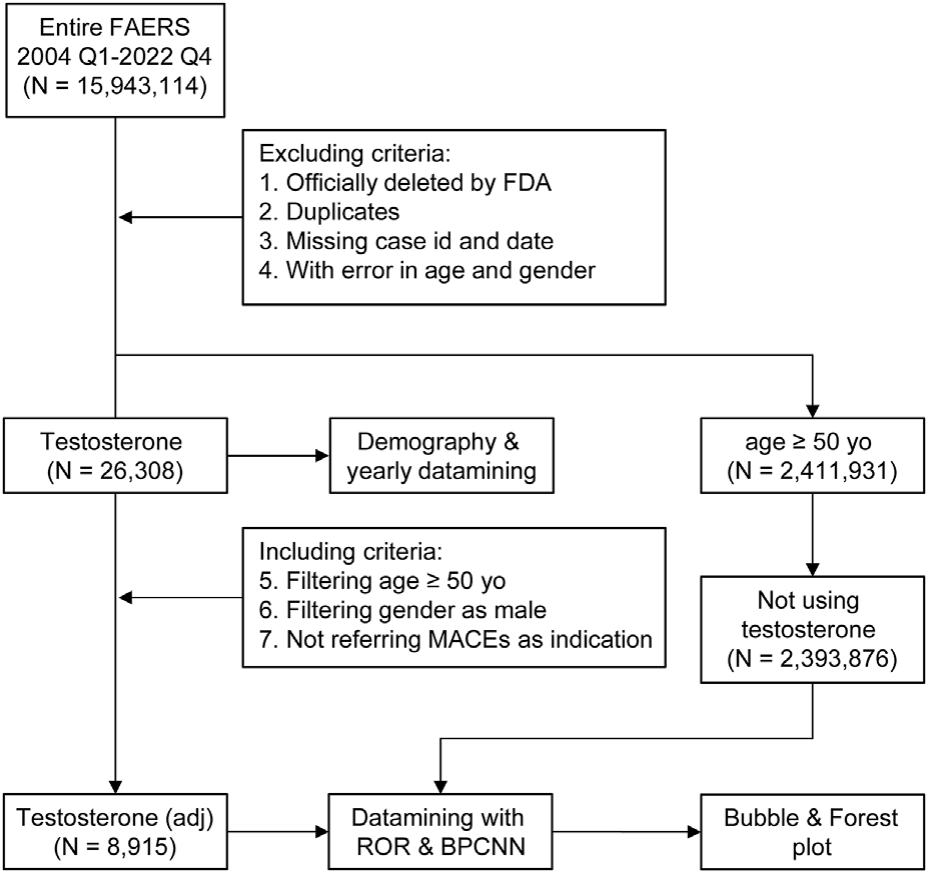
Scheme of the study with the including/excluding criteria. Publicly available FAERS data from 1 January 2004 to 31 December 2022 were filtered using this including/excluding criterion. N, case number of each drug or control group; testosterone stands for reports referring to testosterone treatment (TT), the dataset of TT; testosterone (adj) stands for reports referring to TT without major adverse cardiovascular events (MACEs) as indications and concomitants that were able to generate MACE signals.

Publicly available FAERS data from 1 January 2004 to 31 December 2022 were downloaded as raw data. Criteria of exclusion were demonstrated in the scheme of the study (Figure 1): all reports that were officially deleted by the FDA authority, duplicated, missing case ID and date, or with inaccurate data for gender and age were removed. The data process was conducted using SPSS version 19.0 (Statistical Product and Service Solutions) and R Studio 4.1.2 (R Studio), using a logistic regression model.

### 2.2 Definition of TT and control groups

RxTerms, which is maintained by the US National Library of Medicine, was used as the dictionary database of drug names (Nelson et al., 2011), and the dataset of TT was composed of all reports referring to generic and brand names of testosterone, which were further filtered with testosterone as the primary suspected (PS) drug, and was used for description and statistical analysis later on. The dataset of TT was further stratified into three subgroups according to age, which were non-age [TT: 0–17 years old (yo)], adult (TT: 18–49 yo), and elderly (TT >50 yo). All cases referring to men above 50 yo, without records of TT and without indications referring to MACEs, who were probably undergoing a decrease in their testosterone levels (Petering and Brooks, 2017; Bhasin et al., 2018; Grober et al., 2021), were extracted to represent the population with TD (or with low-T) but without TT (NT >50 yo), as FAERS data do not offer any information about the testosterone level. All cases with the age above 65 yo among the low-T population were further extracted to represent the population with an extended low-T status (NT >65 yo). The dataset of TD was composed of all reports with indications referring to testicular dysfunction, hypogonadism, male andropause, and other preferred terms (PTs) (Supplementary Table S1, indications) provided by the Standardized MedDRA Queries (SMQs) (MedDRA version 23.0) (Katsuhara and Ikeda, 2021), which could logically result in low-T. All concomitant drugs were extracted and evaluated for their association with MACEs, as well as the top 10 indications alongside TT, which could be considered as morbidities. Reports referring to concomitant drugs that could generate valid pharmacovigilance (PV) signals of MACEs or indications referring to MACEs were dropped, and the cleansed dataset of TT was subjected to a data mining procedure to get the adjusted ROR. The datasets of the control groups including TD, NT >50 yo, and NT >65 yo, and morbidities and other risk factors mentioned previously were also calculated for their association with MACEs; a bubble map was created to demonstrate the panorama of interfering effects. The intensity of the PV signal was defined using a value of the lower limit of the information component (IC_025_) and was demonstrated with the size of the bubble. Chi-square (Chi^2^) tests were used to compare patterns of signals generated by different risk factors and assess whether the differences observed are statistically significant.

### 2.3 Definition of MACEs

MACEs were defined using International Classification of Diseases, Clinical Modification, Tenth Revision (ICD-10-CM) diagnosis codes, including cardiovascular death, non-fatal myocardial infarction, and non-fatal stroke (Bosco et al., 2021; Zhang et al., 2021), including acute myocardial infarction (AMI)-induced left heart failure (LHF), ventricular fibrillation (VF)-induced sudden death, and cordial arrhythmia induced by valvular diseases, myocardiopathy, and myocarditis. Thus, MACEs can be detailed as all of the preferred terms containing the keywords “card,” “heart,” “ventricular,” “cereb,” “brain” coupled with “infarction,” “stroke,” “death,” “itis,” “pathy,” “failure,” and “fibri,” which were determined by the Standardized MedDRA Query (version 23.0) terminology (Katsuhara and Ikeda, 2021) (Supplementary Table S1, MACEs). The PTs mapped in “Embolic and thrombotic events (SMQs)” were also used to screen for pharmacovigilance signals.

### 2.4 Descriptive analysis and demography

Qualified reports that underwent exclusion criteria described in Section 2.1 were stratified by gender, age, reporting year, TTO, outcomes, AEs, concomitant drugs, and indications to investigate the demographic profile of testosterone users, especially for reports referring to MACEs. The TTO of each MACE, which could generate PV signals when associated with testosterone, was demonstrated by a box plot. Due to lack of information about testosterone levels in AE reports, age is a notable factor for indicating TD among the male population. The dataset of TT was stratified into subgroups according to the age including 0–9 yo, 10–17 yo, 18–29 yo, 30–49 yo, 50–64 yo, 65–75 yo, 76–85 yo, and above 86 yo. Reports referring to testosterone users from 0–17 yo, 18–49 yo, and above 50 yo were subjected to statistical analyses, and a bubble map of PV signals generated by TT–MACE pairs was plotted. Chi^2^ tests were induced to compare the differences between each age group.

### 2.5 Statistical analysis and signal detection

The data mining procedure using the ROR method was introduced to investigate the disproportionality in reporting the ratio caused by interested drug–AE pairs compared with random drug–AE pairs (detailed in previous publications (Min et al., 2018; Moreland-Head et al., 2021)), which were tandem in with the BCPNN method introduced by Bate et al. (1998), deducing the linkage between the target drug and event by a prior possibility. The association among risk factors including age, morbidities such as diabetes mellitus (DM), and MACEs was also investigated. For the ROR, a positive signal was determined as the count of the targeted drug–AE pair (a) more than three, plus the value of the ROR higher than 1 and the lower limit of the 95% confidence interval (95% CI) exceeding 1. For the BCPNN, a valid PV signal was defined as the value of the lower limit of the information component (IC_025_) exceeding 0, that is, to be specific, the IC_025_ value between 0 and 1.5 was defined as a weak signal, while the IC_025_ value between 1.5 and 3 was considered as a medium signal, and the IC_025_ value >3 was considered as a strong signal.

To demonstrate the developing trend of PV signals generated by TT–MACE pairs, myocardial infarction was picked as an example of testosterone-induced MACEs and was subjected to the calculation of the natural logarithm value of the ROR (ln ROR) and IC_025_ annually with two approaches, including calculations based on the reports of each single year separately and on reports accumulated over time on a yearly basis, mimicking the accumulation of AE reports in the FAERS.

## 3 Results

### 3.1 Demography of TT-associated MACEs

**FIGURE 2:**
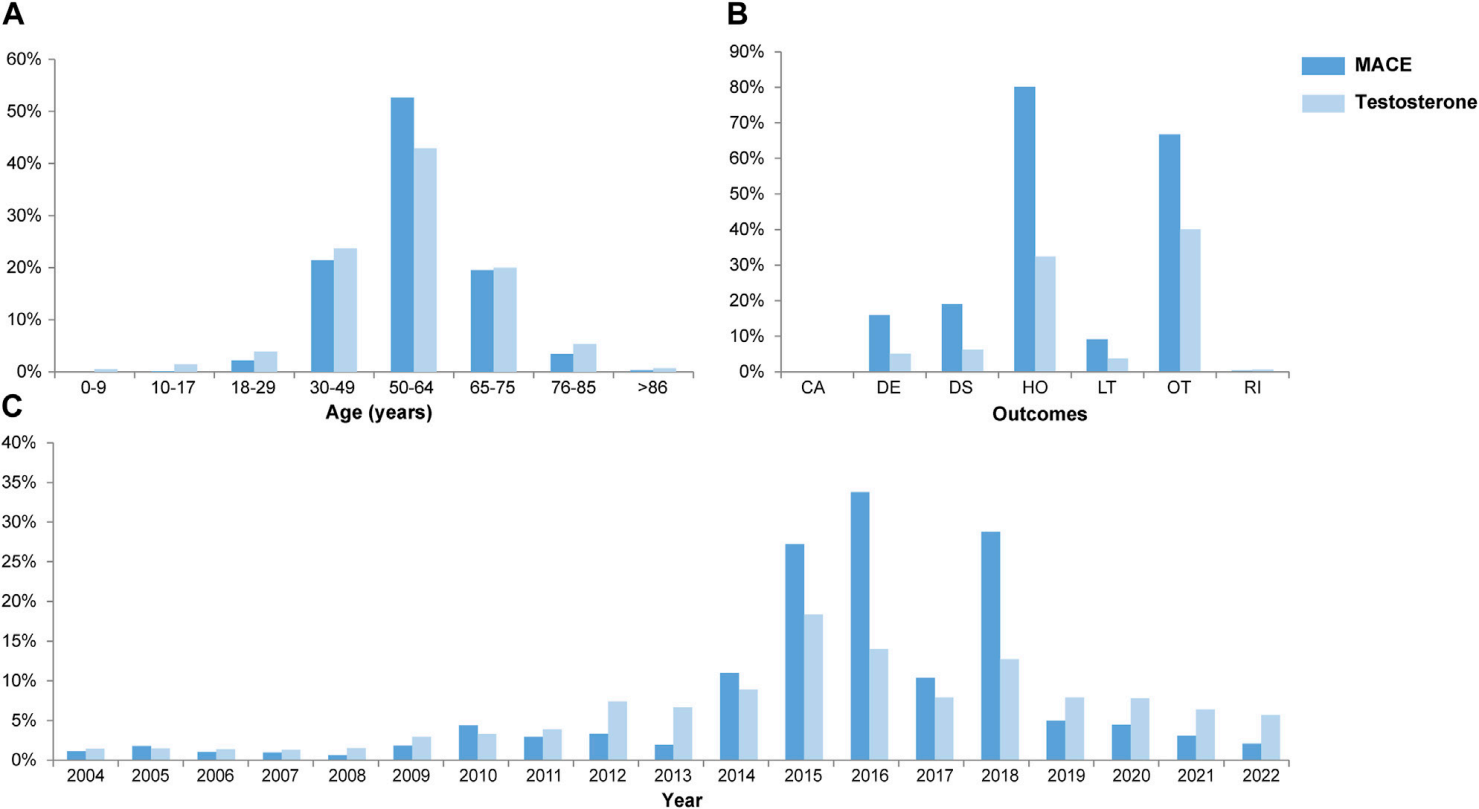
Combined box plots of the demography, including age **(A)**, outcomes **(B)**, and yearly report number **(C)**. *x*-axis, variables; *y*-axis, percentage of concerned variables; years, years old; CA, congenital anomaly; DE, death; DS, disability; HO, hospitalization (initial and prolonged); LT, life threatening; OT, other serious conditions; RI, required intervention.

**FIGURE 3:**
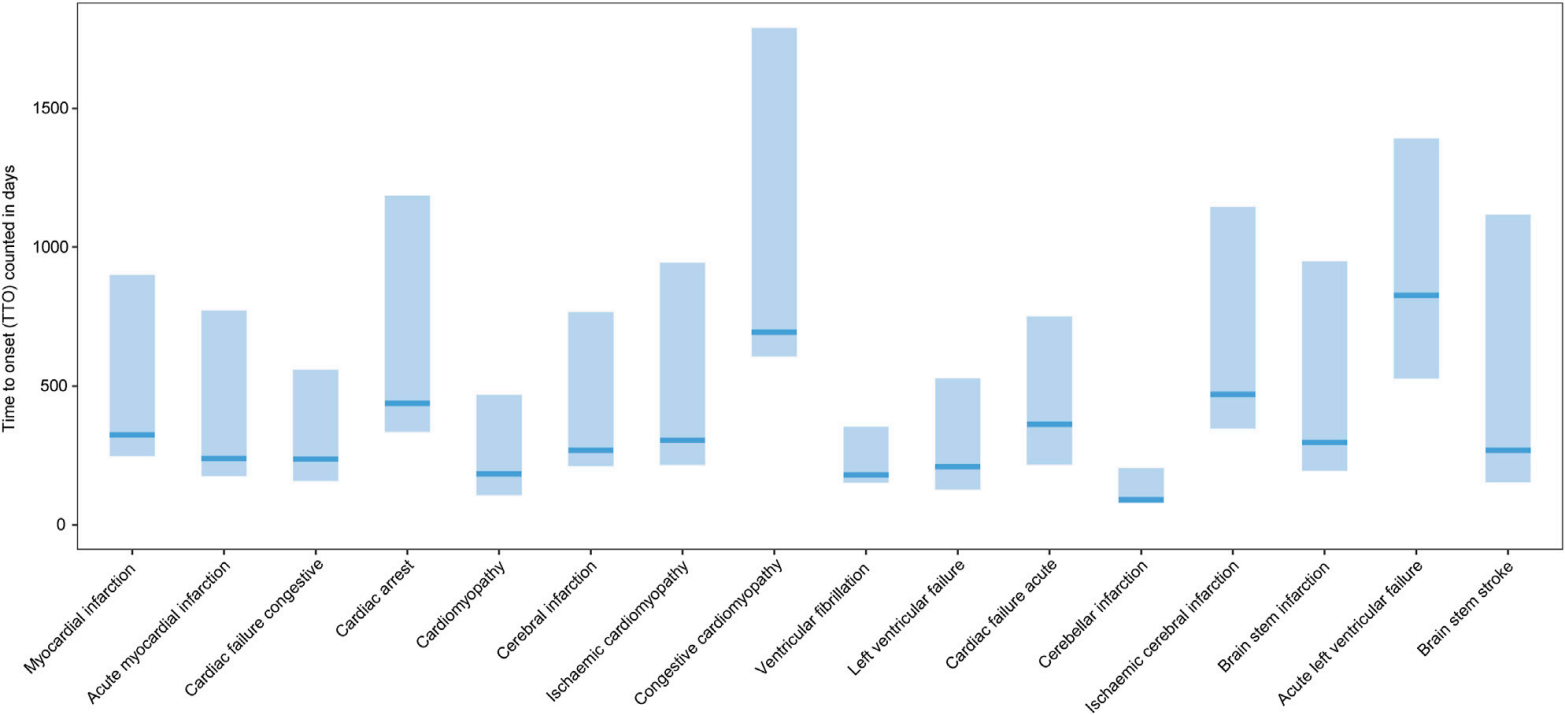
Box plot of the time-to-onset of the MACE. *x*-axis: MACEs; *y*-axis: time-to-onset (TTO) counted in days; bold bar within the stick: median TTO of concerned MACEs; lower end of the stick: 1/4 quantile of the TTO; upper end of the stick: 3/4 quantile of the TTO.

From 1 January 2004 to 31 December 2022, a total of 15,943,114 valid cases were retrieved as raw data. The dataset of TT was composed of 28,921 cases, which referred to testosterone as the PS drug and was used for specific indications, among which 3,057 cases (10.57%) referred to MACEs and 19,727 cases (68.30%) referred to the gender as male of age above 50 yo (Supplementary Table S1). The elderly groups with no exposure to exogenous testosterone with ages above 50 yo (NT >50 yo) and 65 yo (NT >65 yo), accounted for 2,393,876 (Figure 1) and 1,350,931 cases in total, respectively. Men accounted for 97.81% of MACE-related cases, while 90.97% testosterone recipients were male. About 52.60% subjects referring to MACEs were within the age gap of 50 yo–64 yo, while 42.85% of testosterone users were in the same age group (Figure 2A). Notably, most of the MACE-related reports (3,401) were filed between 2014 and 2018 (Figure 2C), which indicated that there must be follow-up cases, as the sum of the number of reports exceeded the total case number of 3,057 and enabled the calculation of the TTO (Supplementary Table S1; Figure 3). The most recorded MACEs included MI (1,700 cases, 55.61%, with a median TTO of 246 days), AMI (605 cases, 19.79%, with a median TTO of 173 days), and cardiac congestive failure (333 cases, 10.79%, with a median TTO of 156 days). There were indications and concomitant drugs that indicated morbidities other than TD, including hypertension, DM, and increased blood cholesterol as the top three indications, while aspirin, lisinopril, and metformin were the top three concomitant drugs. The outcomes of MACE-related reports tended to be more serious than the reports of testosterone users (Figure 2B). There were 2,451 cases (80.18%) of recorded hospitalization or prolonged hospitalization (HO), 581 cases (19.01%) of recorded disability (DS), 489 cases (16.00%) of recorded deaths (DEs), and 2,039 cases (66.70%) referring to other serious conditions (OT).

### 3.2 Adjusted ROR of MACEs

**FIGURE 4:**
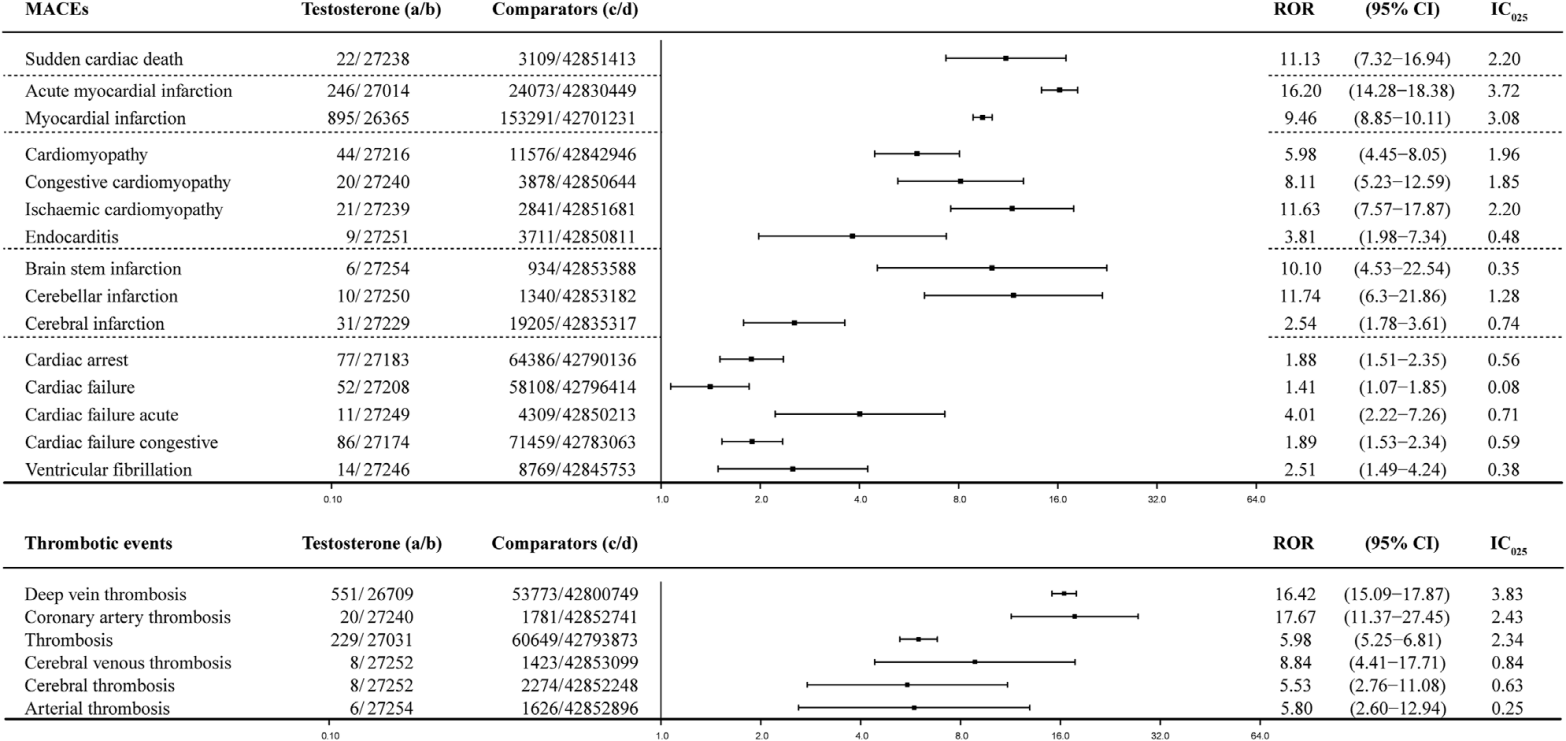
Forest plot of the adjusted ROR of testosterone: dataset of testosterone treatment (TT), which was filtered with the gender as male, age above 50 yo, and all AE reports referring to interfering concomitants and morbidities were dropped prior to the calculation. MACE, major adverse cardiovascular event; a: number of reports referring to both the targeted drug and the interested adverse event (testosterone–MACE); b: number of reports referring to the targeted drug paired with all the reported adverse events (AEs) other than MACEs; c: number of reports referring to MACEs concerning all the other drugs other than the targeted drug; d: number of reports referring to all the reported drug–AE pairs other than testosterone–MACE; 95% CI, 95% confidence interval; IC_025_, lower limit of the information component of the Bayesian confidence propagation neural network.

**FIGURE 5:**
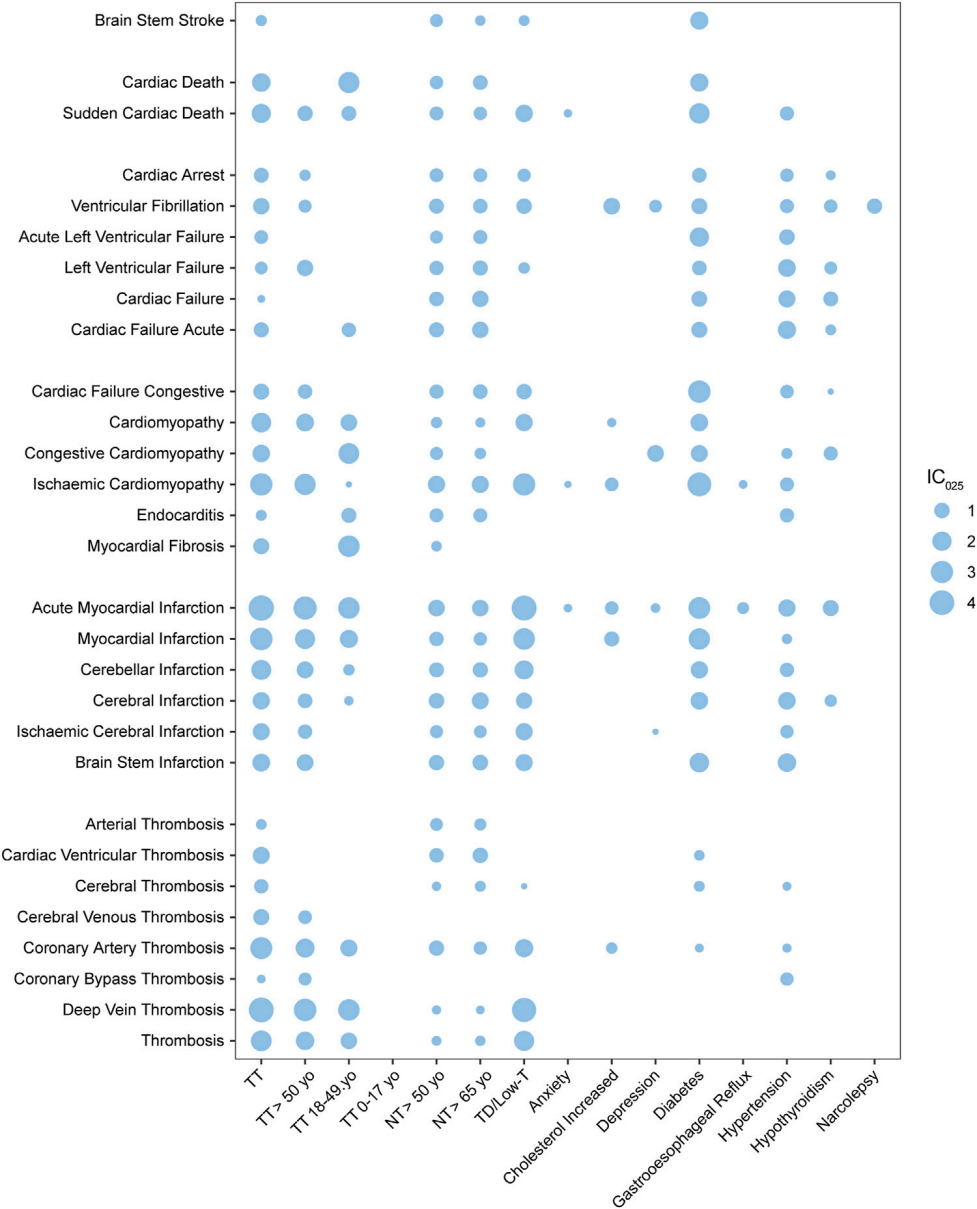
Bubble plot of pharmacovigilance signals generated by MACEs paired with various risk factors; TT, testosterone treatment; NT, no record of TT; TD, testosterone deficiency; low-T, low testosterone level; yo, years old; the intensity of the signal is defined using the value of the lower limit of the information component (IC_025_) and was demonstrated with the size of the bubble.

Paired with TT, MACEs generated various PV signals that could be roughly categorized into four subgroups: infarctions, cardiac failure, cardiomyopathy, and cardiac arrhythmia, such as VF and cardiac arrest (Figure 4). Infarctions were the most significant PV signals generated when paired with testosterone, including AMI with the ROR value as 16.20 (95% CI: 14.28–18.38) and IC_025_ as 3.72 and MI with the ROR value as 9.46 (95% CI: 8.85–10.11) and IC_025_ as 3.08; both were strong signals generated by the BCPNN. Infarctions related to the brain were relatively weaker, including cerebellar infarction with the ROR value as 11.74 (95% CI: 6.30–21.86) and IC_025_ as 1.28. Cardiac infarctions were considerably more common and more intense in signals on the IC_025_ basis, although the ROR value was roughly at the same level. There were 895 cases of MI and 246 cases of AMI, while cerebral infarctions were counted in dozens. Cardiomyopathy-related AEs and sudden cardiac deaths were the next category, counted in dozens but generated medium signals, including sudden cardiac deaths with the ROR value as 11.13 (95% CI: 7.32–16.94) and IC_025_ as 2.20; cardiomyopathy with the ROR value as 5.98 (95% CI: 4.45–8.05) and IC_025_ as 1.96; congestive cardiomyopathy with the ROR value as 8.11 (95% CI: 5.23–12.59) and IC_025_ as 1.85; and ischemic cardiomyopathy with the ROR value as 11.63 (95% CI: 7.57–17.87) and IC_025_ as 2.20. To interpret the results, we also investigated thrombosis events, due to the reasonable speculation that infarctions and strokes might be rooted in embolic and thrombosis events**.** Arterial thrombosis and cerebral thrombosis counted less than 10 cases, while coronary arterial thrombosis count 20 cases, with the ROR value as 17.67 (95% CI: 11.37–27.45) and IC_025_ as 2.43. Cardiac failure and cardiac arrhythmia events, such as VF, were rare and weak, among which cardiac failure acute was the strongest signal with the ROR value as 4.01 (95% CI: 2.22–7.26) and IC_025_ as 0.71. Although ventricular arrhythmia, such as VF, in itself is not a direct cause of left ventricular cardiac failure, it can lead to it if it is not treated promptly (Packer, 1992), including VF with the ROR value as 2.51 (95% CI: 1.49–4.24) and IC_025_ as 0.38. Cardiac arrest was the most common AE of arrhythmia counting 77 cases, with the ROR value as 1.88 (95% CI: 1.51–2.35) and IC_025_ as 0.56. Compared with crude IC_025_ (Figure 5, detailed in Supplementary Table S2), almost all the signals were weaker for the adjusted ROR, including AMI with the IC_025_ value as 4.25 (crude) vs. 3.72 (adjusted); MI with the IC_025_ value as 3.12 (crude) vs. 3.71 (adjusted); and ischemic cardiomyopathy with the IC_025_ value as 3.02 (crude) vs. 2.20 (adjusted), while weak signals such as cardiac deaths, brain stem strokes, and brain stem infarctions disappeared for the adjusted calculation, due to the casting out of reports referring to indications of MACEs and concomitant drugs that could have generated valid PV signals of MACEs.

### 3.3 Panorama of risk factors and MACEs

As shown in the bubble map (Figure 5, detailed in Supplementary Table S2), the value of IC_025_ was represented by the size of the bubble, while every bubble for a TT–MACE combination was left unpainted if the combination did not generate valid PV signals. The *x*-axis demonstrated risk factors including TT (alongside the subgroups of TT: 0–17 yo, TT: 18–49 yo, and TT >50 yo), elderly without TT (including NT >50 yo and NT >65 yo), TD, and top 10 indications alongside TT except “pain,” while the *y*-axis listed MACEs with which they were paired. The PTs of MACEs were roughly categorized into five subgroups: infarction and stroke, cardiac death, cardiomyopathy, cardiac arrhythmia, and cardiac failure. The PTs related to embolic and thrombosis events were also analyzed and demonstrated in Figure 5.

#### 3.3.1 Morbidities

Morbidities including TD, DM, and hypertension were generated and shared almost all MACE-related PV signals with TT, while the other eight indications, depression, anxiety, gastroesophageal reflux disease, narcolepsy, and other morbidities, were not so associated with MACEs. Compared with TT, DM as a risk factor demonstrated notable figures when associated with MACE, including brain stem stroke (BSS) with the IC_025_ value as 1.65 (vs. the IC_025_ value as 0.29 for TT–BSS), acute left ventricular failure (LVF) with IC_025_ as 2.00 (vs. 0.65 for TT–LVF), and cardiac failure (CF) with IC_025_ as 3.05 (vs*.* 1.10 for TT–CF); hypertension–LVF generated the IC_025_ value as 1.5, but DM and hypertension generated little thrombotic PV signals. Cardiac death, endocarditis, and myocardial fibrosis were unique to TT and generated PV signals with the IC_025_ value as 1.77, 0.28, and 1.13, respectively, compared with hypertension and TD.

#### 3.3.2 Thrombotic events

While testosterone was found to increase the risk of venous thrombotic events, the risk of arterial thrombotic events, particularly the events related to the heart or brain did not exhibit a significant increase. In total, when paired with testosterone, thrombosis generated medium signals with IC_025_ as 2.47 (vs. 2.25 for TD–thrombosis), while coronal arterial thrombosis (CAT) generated IC_025_ as 2.92 (vs. 1.68 for TD–CAT) and deep vein thrombosis (DVT) with IC_025_ as 4.04 (*vs.* 3.76 for TD–DVT). When it comes to cardiac- or cerebral-related thrombosis, cardiac ventricular thrombosis has a value of 1.36, while cerebral thrombosis has a value of 0.78 and cerebral venous thrombosis has a value of 1.15. As an endogenous hormone, testosterone is widely used for TT (Baillargeon et al., 2015; Elliott et al., 2017), and there is limited evidence that other medications can deal with low-T. It was unlikely that we could distinguish the differences between the datasets of TD and TT. However, for arterial thrombosis, cardiac ventricular thrombosis, cerebral thrombosis, and coronal artery thrombosis, there were significant differences between TT and TD, which demonstrated almost no association; while for deep vein thrombosis, there was no significant difference between TT and TD.

#### 3.3.3 Age and MACEs

Normal aging is a risk factor for various health hazards, including hypogonadism, CV issues, and MACEs (Nguyen et al., 2015). Compared with morbidities including TD, DM, and hypertension, the risk factor of age is insignificant, based on FAERS data. Weak PV signals could be generated between an elderly group without TT (NT >50 yo and NT >65 yo) and arterial thrombosis events and cardiac failure events, while TD could not (Figure 5). For lethal cases, when associated with NT >50 yo, sudden cardiac death generated the IC_025_ value of 0.75, while the elderly group of TT (TT >50 yo) generated the IC_025_ value of 0.98; meanwhile, the TD–sudden cardiac death pair generated the IC_025_ value of 1.48. Cardiac death associated with TT could generate a medium signal, but it can later be ruled out by the adjusted ROR, mentioned in Section 3.2. For testosterone users, most MACE signals were stronger in the age group above 50 yo (TT >50 yo) than in the age group of 18–49 yo (TT 18–49 yo), especially for ischemic cardiomyopathy (IC_025_ as 2.72) and cerebral-related infarctions, including ischemic cerebral infarction (IC_025_ as 0.78) and brain stem infarction (IC_025_ as 1.31). However, for congestive cardiomyopathy (IC_025_ as 2.45), endocarditis (IC_025_ as 0.92) and myocardial fibrosis (IC_025_ as 2.72), which are unique to testosterone users, have stronger signals when associated with TT 18–49 yo, compared to TT and TT >50 yo. The signals generated by TT subgroups stratified by age were generally weaker than TT. MACEs and thrombosis events generated no valid PV signals in the non-age group (TT: 0–17 yo).

#### 3.3.4 The chi-square test

Chi square (Chi^2^) tests were applied to investigate correlations between the patterns of MACEs associated with various risk factors, including TD, with or without TT, and morbidities, using a null hypothesis claiming that the prevalence of any two given series of IC_025_ was the same (Supplementary Table S3). No significant differences were found based on the Chi^2^ test, but among all the series, testosterone users aged between 18 and 49 yo (TT: 18–49 yo) exhibited a slight deviation from testosterone users aged above 65 yo (*p* value = 0.54) and TD (*p* value = 0.4) and non-TT cases above 50 yo (*p* value = 0.79) and above 65 yo (*p* value = 0.75).

### 3.4 Developing trend of PV signals

**FIGURE 6:**
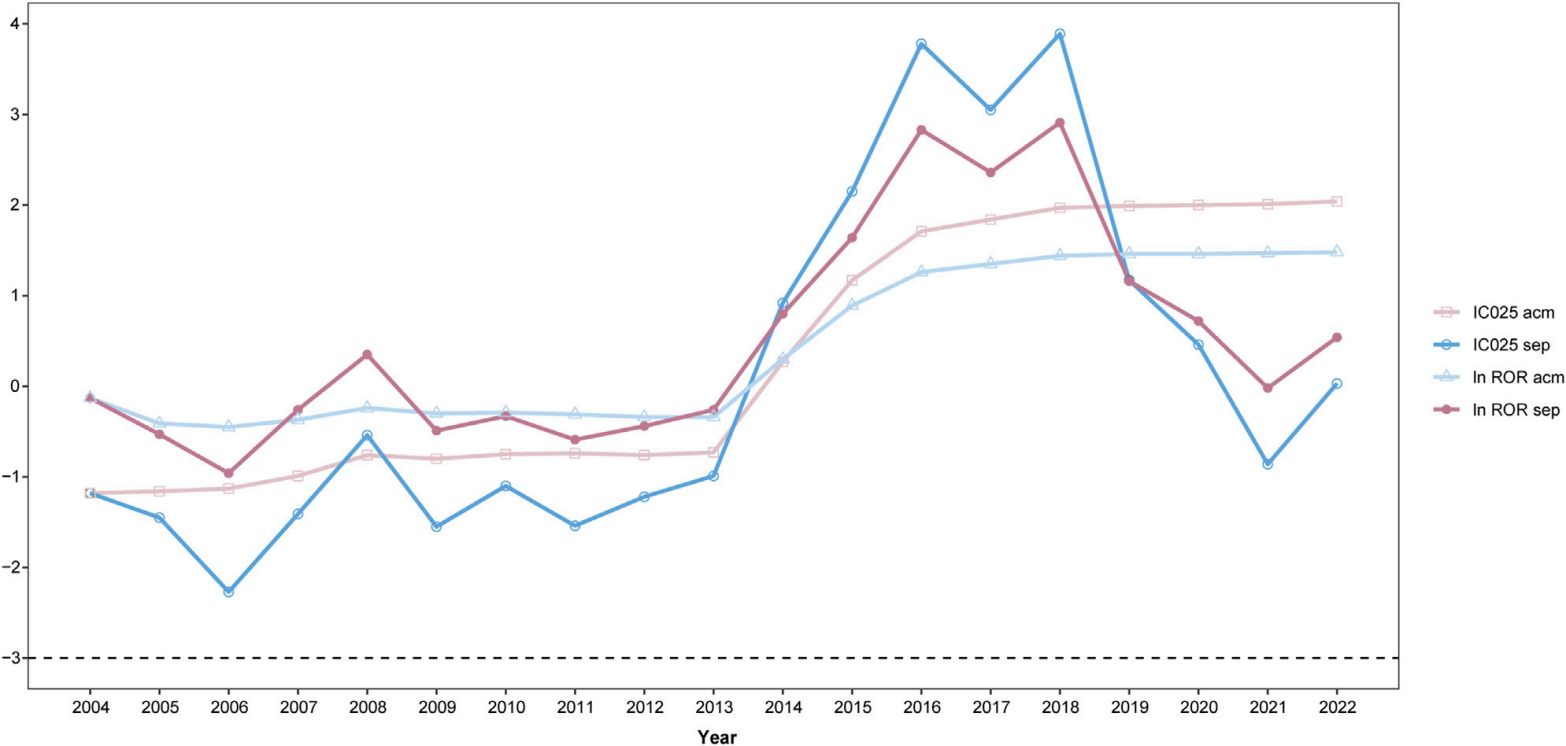
Trend of pharmacovigilance signals taking myocardial infarction (MI) as an example; IC_025_, lower limit of the information component; ln ROR, natural logarithm value of the ROR; acm, calculation based on the accumulated dataset year after year; sep, calculation based on the dataset of every single year; *x*-axis, time in the calendar order; *y*-axis, natural algorithm value of the ROR (ln ROR); IC_025_, lower limit of the information component.

Data mining procedures were also applied to investigate the association between TT and MACEs based on the reports of each year separately and on the dataset that accumulated over time by adding yearly data to the previous years. To demonstrate the changing pattern of the yearly tendency (Figure 6, detailed in Supplementary Table S4, taking MI as an example), the natural logarithm value of the ROR (ln ROR) and IC_025_ was used as vertical coordinates and were plotted against each calendar year as horizontal coordinates. For IC_025_, there was no valid PV signal of MI for both yearly calculations, until 2014; while for ln ROR, there was a valid PV signal in 2008. Since 2014, the value of ln ROR and IC_025_ changed dramatically. For the accumulation calculation, the signal achieved equilibrium since 2019; while for the separate calculation, the plot demonstrated a double peak and then underwent a decrease since 2019.

## 4 Discussion

It should be noted that the TRAVERSE. (2019) began in 2018, which was the first TT trial designed to fully evaluate testosterone-induced CV events. The results of this trial, which were derived from a double-blind and placebo-controlled study, would provide more conclusive evidence of the CV safety of TT, but the findings might not be available for a decade (Bhasin et al., 2022). Our work is the latest data mining approach used for investigating the association between TT and MACEs based on the FAERS database.

### 4.1 Control group

The testosterone level is usually not shown by AE reports in the FAERS, although the dosage information might give us some clues about severity of TD, as the therapeutic dosage was intended to maintain the suggested threshold level between 300 and 350 ng/dL according to the guidelines (Salter and Mulhall, 2019). In addition, testosterone is widely used for men with hypogonadism as TRT (Elliott et al., 2017; Bhasin et al., 2018), which meant that the population of testosterone users largely overlapped with the population with TD. Therefore, it was challenging to define a control group with TD and without TT. All the cases referring to men above 50 yo (and above 65 yo), without recording TT and without indications referring to MACEs, who were probably undergoing a decrease in testosterone levels (Petering and Brooks, 2017; Bhasin et al., 2018; Grober et al., 2021) (Supplementary Table S3), were extracted to represent the population of low-T and served as control groups together with the TD group, which was composed of all reports referring to low-T as indications. Signals generated by testosterone paired with MACEs and by control groups paired with MACEs proved that the dataset of TT and TD were largely composed of the same cases, and most of the cases of TT were indeed cases of TRT. Testosterone users aged between 18 and 49 yo demonstrated a slight deviation from control groups including TD, non-TT cases, and testosterone users aged above 50 yo, which indicated that age is a significant risk factor of MACEs, as the low-T status is a risk factor for MACEs (Shores et al., 2012). Previous publications stated that testosterone could improve the blood fibrinolytic activity of patients, thus playing a role in the treatment of occlusive vascular diseases, but its causal relationship was worth considering (Fearnley and Chakrabarti, 1962). If the lower testosterone status was amended by TT, patients aged between 18 and 49 yo achieved benefits for preventing MACEs, especially for congestive cardiomyopathy, endocarditis, and myocardial fibrosis, which were almost unique to patients receiving TT between 18 and 49 yo.

### 4.2 Interfering

TD could be caused by various conditions, including normal aging, hypogonadism, injury, or dysfunction of testicles, disorders that affect the pituitary gland and cause kidney diseases (Bhasin et al., 2018). Since not all TT was associated with a decreased mortality compared with no TT (Shores et al., 2012) and DM and hypertension also generated valid PV signals referring to MACEs, our findings should be interpreted cautiously, considering that morbidities and concomitant drugs may be a source of bias. To eliminate interfering caused by concomitant drugs and indications, the dataset which was extracted from the FAERS underwent the data mining analysis described in Section 2.5. Reports that referred to concomitant drugs that generated valid PV signals and indications related to MACEs were cast out from the dataset of TT, and the adjusted ROR was calculated (Supplementary Table S1). Although all the values of the adjusted ROR and IC_025_ were lower than the crude calculation, it indicated that lesser cases were taken into consideration, but with more credibility.

TT might be beneficial to TD patients as it reduces cardiac failure and cardiac arrhythmia; however, it increased the risk of infarctions and cardiomyopathy, according to FAERS data. The mechanism might show that androgen exposure increases cardiotoxicity by activating inflammatory mediators (Sessa et al., 2022) and that testosterone increased the risk of thrombosis, and infarctions could be caused by thrombosis, which matched with the result of Luo’s study, stating that genetically predicted endogenous testosterone is positively correlated with thromboembolism and heart failure, especially in men (Luo et al., 2019). Another systematic review and meta-analysis of placebo-controlled randomized trials on men, who had been treated with testosterone for more than 12 weeks and who reported CV events, concluded that exogenous testosterone increased the risk of CV events (Xu et al., 2013). We made a comprehensive summary of the association between TT and CV events (Gagliano-Jucá and Basaria, 2019) and mentioned that there were several retrospectives and prescription database studies referring to the increased number of CV events in men who received TRT (Vigen et al., 2013; Finkle et al., 2014; Etminan et al., 2015; Martinez et al., 2016).

In addition, cardiomyopathy-related signals could be generated when associated with TT, and stronger signals than those associated with TD could also be generated, when evaluated by IC_025_. Cardiomyopathy is usually caused by the long-term use of anabolic steroids, which are synthetic derivatives of testosterone, and due to the toxicity of other medications (Liang et al., 2019), they might cause the irreversible destruction of the heart tissue (Salimi et al., 2020). Our findings indicated that TT notably increases the risk of cardiomyopathy and other types of damage referring to the heart, such as endocarditis and myocardial fibrosis, and this could be supported by a case report that claims that exogenous testosterone is a rare and reversible cause of cardiomyopathy in young and otherwise healthy athletes, and a high index of suspicion is required to prevent potentially fatal side effects (Doleeb et al., 2019). Congestive cardiomyopathy tends to occur among testosterone users aged 18–49 yo, while ischemic cardiomyopathy tends to occur among patients above 50 yo. All cardiomyopathy-related events generated weaker signals among subjects above 50 yo and 65 yo who had no record of TT, compared to their counterparts who accepted TT. Therefore, a conservative use of testosterone is warranted in men with CV diseases, who may be at greater risk for adverse outcomes (Shores and Matsumoto, 2014).

There were several studies that suggested that testosterone therapy was not significantly associated with the occurrence of MACEs or even reduced such risks (Gencer and Mach, 2016; Maggi et al., 2016), which can also be explained by our study, considering the morbidities. Normal aging, TD, DM, and hypertension were morbidities that were associated with MACE; notably, they seemed to have little effect on thrombosis events, except in TD (Figure 5). Brain stem stroke, ischemic cardiomyopathy, sudden cardiac death, and cardiac failure-related events associated with diabetic patients generated stronger signals and were associated with TT, while cardiac failure-related events associated with hypertension generated strong signals than those associated with TT. These findings indicated that TT might benefit the patient with DM and hypertension by preventing cardiac failure, especially cardiac failure events such as left ventricular failure and acute left ventricular failure, which were more related to cardiac arrhythmia, such as VF. Although VF itself is not a direct cause of left ventricular cardiac failure, it can lead to it if it is not treated promptly (Packer, 1992). In general, MACE-related signals generated by TT were stronger than those generated by TD, but were weaker than their counterparts generated by patients with DM and hypertension. These findings indicated that TT may improve the cardiac output, as suggested by previous publications (Pugh et al., 2004; Park et al., 2021), potentially benefiting ischemic myopathy. However, it may have a negative impact on cardiac failure caused by congestive reasons, indicating that the effects of testosterone on the heart are complex and context-dependent; while it seems to have no significant effect on cardiac arrhythmia-related MACEs, although animal models suggest that androgen increases the risk of hydro-electrolytic and autonomic imbalances, but did not alter the vascular or cardiac function or morphology (Salimi et al., 2020). Notably, the PT of cardiac death generated a valid PV signal when associated with TT by a crude calculation, but was ruled out from the results of the adjusted ROR, which indicated that this signal might be contributed by interfering, but is still worthy of demonstration in the bubble map.

### 4.3 Underreporting

As an endogenous hormone, testosterone was first used for TT in the late 1930s and has become an established treatment for male hypogonadism, since then (Bhasin et al., 2007); however, reports referring to TT-induced MACEs are relatively rare in the FARES, and PV signals of TT-induced MACEs did not emerge until 2015. To make the situation even more precarious, in 2014, the FDA agreed that enough attention should be paid to the potential risk signals of CV risks (Garnick, 2015; Seftel, 2015). Therefore, we should be more cautious when we monitor PV signals in a retrospective study on the signals which were dramatically increased, and then in the next few years, it gradually decreased. Hypothetically, if there is an association that exists between the risk factor and the outcome, the disproportionality caused by it should have presented us with some stable levels on a yearly basis. To fix the biases inherent to PV studies and notices publicized by authorities, AE reports filed to the FAERS in the following several years should be carefully examined and subjected to more restricted inclusion criteria, such as only accepting the reports filed by health professionals. We shall also focus on unreported signals, which were steady and gradually increased for the pharmacosurveillance purpose. These findings also indicated that there might still be a large number of related adverse events that went unrecorded, and the health workers should be reminded to pay more attention to MACEs associated with TT.

### 4.4 Limitations

There were several concerns that might undermine the credibility of this paper. Although the dataset of TD was largely overlapped with the dataset of TT, there were 5,072 cases (17.54%) of TT referring to indications as the “product used for unknown indication.” Therefore, for these cases, the possibility of alternative testosterone treatment other than TRT, for example, using as bodybuilding supplements (Doleeb et al., 2019), cannot be ruled out. However, we cannot cast out these cases either. Spontaneous reporting systems including the FAERS were exposed to the biases inherent to PV studies. The FDA Advisory Committee agreed that enough attention should be paid to the potential risk signals of CV risks in 2014 (Garnick, 2015; Seftel, 2015)**,** which coincidently matched the gush of MACE-related ADR reports and a surge in the ROR for the TT–MACE pair. Therefore, we should be cautious when we try to interpret the calculations. In addition, since TT is the prevailing treatment for TD, it is unlikely that we could discuss testosterone-induced MACEs without considering that these conditions might be caused by a low testosterone status (Shores and Matsumoto, 2014), and the causal relationships between MACEs and testosterone could not be confirmed by the data mining approach alone.

## 5 Conclusions

In conclusion, we aimed to address the long-standing controversy shrouding the association between TT and MACEs, based on FARES data. Age turned out to be the most significant risk factor for TT-induced MACEs. Compared with the TD population, TT increased the risk of MACEs, especially for infarction- and cardiomyopathy-related events, while it demonstrated curative effects when paired with cardiac arrhythmia- and cardiac failure-related events and stroke and sudden cardiac death. TT demonstrated benefits in preventing MACEs related to cardiac congestive failure and ischemic events for patients who suffered with DM and hypertension. Endocarditis and myocardial fibrosis were uniquely associated with TT, especially among male adults. Computational studies are crucial in setting up well-designed scientific studies, while micro-RNA molecules have been shown to play various significant roles in many physiological and pathophysiological processes in living organisms (Lukasik and Zielenkiewicz, 2019). We reported that micro-RNAs, including hsa-miR-133a, hsa-miR-21, hsa-miR-499a, hsa-miR-1, and hsa-miR-126, played an active role in the genesis and development of different types of heart damage, using an integrative analysis (Sessa et al., 2018). Further studies focused on identifying molecular markers related to MACEs that could be used for both diagnostic and therapeutic purposes and could have an ambitious goal of revealing the mechanism of TT-induced MACEs. Healthcare workers should be fully aware of the benefits and possible risks of TT to improve the effectiveness and safety of drug treatments and patients’ quality of life.

## Data Availability

The datasets presented in this study can be found in online repositories. The names of the repository/repositories and accession number(s) can be found in the following: https://fis.fda.gov/extensions/FPD-QDE-FAERS/FPD-QDE-FAERS.html.
