# Supplementary material for: Major adverse cardiovascular events associated with testosterone treatment: a pharmacovigilance study of the FAERS database
Source: Front Pharmacol. 2023 Jul 12;14:1182113. doi: 10.3389/fphar.2023.1182113 (PMC10370495; doi:10.3389/fphar.2023.1182113)
Supplement: Supplementary file 1 [file Table1.docx]

**Supplement 1. Demographic table extend for concomitants, morbidities alongside TT and list of preferred terms of MACEs.**

|  | **MACE** | | **TT** | |
| --- | --- | --- | --- | --- |
|  | **N = 3,057** | | **N = 28,921** | |
| **Gender:** |  |  |  |  |
| Male | 2990 | 97.81% | 26308 | 90.97% |
| Female | 64 | 2.09% | 2583 | 8.93% |
|  |  |  |  |  |
| **Age:** |  |  |  |  |
| 0-9 yo | 0 | 0.00% | 157 | 0.55% |
| 10-17 yo | 2 | 0.07% | 421 | 1.47% |
| 18-29 yo | 66 | 2.17% | 1116 | 3.90% |
| 30-49 yo | 653 | 21.45% | 6797 | 23.73% |
| 50-64 yo | 1601 | 52.60% | 12273 | 42.85% |
| 65-75 yo | 595 | 19.55% | 5728 | 20.00% |
| 76-85 yo | 103 | 3.38% | 1536 | 5.36% |
| >86yo | 9 | 0.30% | 190 | 0.66% |
| mean ± SD | 55.10 ± 14.77 | | 56.55 ± 11.26 | |
| median (quantile) | 57.00 (47.00, 65.00) | | 57.00 (50.00, 64.00) | |
|  |  |  |  |  |
| **MACEs:** |  |  | **Time to onset** (days, quantile) | |
| myocardial infarction | 1700 | 55.61% | 246.00 (78.00, 577.00) | |
| acute myocardial infarction | 605 | 19.79% | 173.00 (69.00, 531.00) | |
| cardiac failure congestive | 330 | 10.79% | 156.00 (80.00, 323.50) | |
| cardiac arrest | 207 | 6.77% | 333.00 (107.00, 746.75) | |
| cardiomyopathy | 105 | 3.43% | 106.50 (75.75, 287.75) | |
| cerebral infarction | 101 | 3.30% | 210.50 (56.75, 501.00) | |
| ischaemic cardiomyopathy | 54 | 1.77% | 214.50 (92.00, 639.75) | |
| congestive cardiomyopathy | 42 | 1.37% | 605.50 (84.50, 1101.75) | |
| ventricular fibrillation | 40 | 1.31% | 150.00 (29.00, 175.00) | |
| left ventricular failure | 24 | 0.79% | 126.00 (84.00, 319.25) | |
| cardiac failure acute | 22 | 0.72% | 215.50 (146.75, 391.50) | |
| cerebellar infarction | 20 | 0.65% | 77.00 (9.00, 119.00) | |
| ischaemic cerebral infarction | 14 | 0.46% | 345.50 (124.00, 677.50) | |
| brain stem infarction | 13 | 0.43% | 193.00 (102.50, 654.50) | |
| acute left ventricular failure | 10 | 0.33% | 525.00 (302.50, 656.50) | |
| brain stem stroke | 6 | 0.20% | 152.00 (113.50, 853.00) | |
|  |  |  |  |  |
| **Outcomes:** |  |  |  |  |
| congenital anomaly | 1 | 0.03% | 8 | 0.03% |
| death | 489 | 16.00% | 1447 | 5.00% |
| disability | 581 | 19.01% | 1784 | 6.17% |
| hospitalization | 2451 | 80.18% | 9367 | 32.39% |
| life-threatening | 277 | 9.06% | 1060 | 3.67% |
| other serious | 2039 | 66.70% | 11557 | 39.96% |
| required intervention | 9 | 0.29% | 149 | 0.52% |
|  |  |  |  |  |
| **Yearly report：** | | |  |  |
| 2004 | 35 | 1.14% | 424 | 1.47% |
| 2005 | 55 | 1.80% | 437 | 1.51% |
| 2006 | 31 | 1.01% | 396 | 1.37% |
| 2007 | 30 | 0.98% | 373 | 1.29% |
| 2008 | 20 | 0.65% | 440 | 1.52% |
| 2009 | 56 | 1.83% | 849 | 2.94% |
| 2010 | 134 | 4.38% | 955 | 3.30% |
| 2011 | 90 | 2.94% | 1127 | 3.90% |
| 2012 | 102 | 3.34% | 2143 | 7.41% |
| 2013 | 59 | 1.93% | 1922 | 6.65% |
| 2014 | 337 | 11.02% | 2571 | 8.89% |
| 2015 | 833 | 27.25% | 5303 | 18.34% |
| 2016 | 1033 | 33.79% | 4051 | 14.01% |
| 2017 | 318 | 10.40% | 2288 | 7.91% |
| 2018 | 880 | 28.79% | 3684 | 12.74% |
| 2019 | 152 | 4.97% | 2287 | 7.91% |
| 2020 | 137 | 4.48% | 2257 | 7.80% |
| 2021 | 94 | 3.07% | 1850 | 6.40% |
| 2022 | 64 | 2.09% | 1649 | 5.70% |

|  | **MACEs:** | **concomitants:** | | **N** | **%** | **Morbidities:** | **N** | **%** | **Indications:** | **N** | **%** |
| --- | --- | --- | --- | --- | --- | --- | --- | --- | --- | --- | --- |
| 0 | myocarditis septic | aspirin | 550 | | 17.99% | hypertension | 426 | 13.94% | blood testosterone decreased | 7880 | 27.25% |
| 1 | eosinophilic myocarditis | lisinopril | | 389 | 12.72% | diabetes mellitus | 247 | 8.08% | product used for unknown indication | 5072 | 17.54% |
| 2 | giant cell myocarditis | metformin hydrochloride | | 278 | 9.09% | blood cholesterol increased | 146 | 4.78% | androgen replacement therapy | 1887 | 6.52% |
| 3 | autoimmune myocarditis | furosemide | | 242 | 7.92% | anxiety | 145 | 4.74% | hypogonadism | 1605 | 5.55% |
| 4 | coxsackie carditis | simvastatin | | 227 | 7.43% | gastrooesophageal reflux disease | 141 | 4.61% | hormone replacement therapy | 1602 | 5.54% |
| 5 | coxsackie myocarditis | clopidogrel bisulfate | | 223 | 7.29% | blood cholesterol | 135 | 4.42% | androgen deficiency | 437 | 1.51% |
| 6 | cytomegalovirus myocarditis | atorvastatin calcium | | 212 | 6.93% | cardiac disorder | 127 | 4.15% | asthenia | 362 | 1.25% |
| 7 | enterovirus myocarditis | metoprolol | | 208 | 6.80% | anticoagulant therapy | 86 | 2.81% | fatigue | 311 | 1.08% |
| 8 | hypersensitivity myocarditis | atorvastatin | | 205 | 6.71% | hypothyroidism | 73 | 2.39% | erectile dysfunction | 306 | 1.06% |
| 9 | immune-mediated myocarditis | omeprazole | | 174 | 5.69% | type 2 diabetes mellitus | 66 | 2.16% | hypogonadism male | 261 | 0.90% |
| 10 | lupus myocarditis | gabapentin | | 173 | 5.66% | insomnia | 64 | 2.09% | libido decreased | 250 | 0.86% |
| 11 | lyme carditis | carvedilol | | 171 | 5.59% | back pain | 64 | 2.09% | testicular failure | 194 | 0.67% |
| 12 | malarial myocarditis | amlodipine besylate | | 171 | 5.59% | hyperlipidaemia | 55 | 1.80% | transgender hormonal therapy | 172 | 0.59% |
| 13 | myocarditis | cholecalciferol | | 168 | 5.50% | blood cholesterol abnormal | 55 | 1.80% | accidental exposure | 155 | 0.54% |
| 14 | myocarditis bacterial | levothyroxine sodium | | 164 | 5.36% | blood pressure abnormal | 53 | 1.73% | blood follicle stimulating hormone decreased | 149 | 0.52% |
| 15 | myocarditis helminthic | rosuvastatin calcium | | 145 | 4.74% | blood pressure measurement | 53 | 1.73% | hypopituitarism | 149 | 0.52% |
| 16 | myocarditis infectious | acetaminophen__hydrocodone bitartrate | | 140 | 4.58% | atrial fibrillation | 51 | 1.67% | drug use for unknown indication | 147 | 0.51% |
| 17 | myocarditis meningococcal | pantoprazole sodium | | 139 | 4.55% | hiv infection | 47 | 1.54% | blood luteinising hormone decreased | 145 | 0.50% |
| 18 | myocarditis mycotic | alprazolam | | 127 | 4.15% | rheumatoid arthritis | 47 | 1.54% |  |  |  |
| 19 | myocarditis post infection | levothyroxine | | 125 | 4.09% | thrombosis prophylaxis | 46 | 1.50% |  |  |  |
| 20 | myocarditis syphilitic |  | |  |  |  |  |  |  |  |  |
| 21 | myocarditis toxoplasmal |  | |  |  |  |  |  |  |  |  |
| 22 | radiation myocarditis |  | |  |  |  |  |  |  |  |  |
| 23 | viral myocarditis |  | |  |  |  |  |  |  |  |  |
| 24 | pericarditis uraemic |  | |  |  |  |  |  |  |  |  |
| 25 | pericarditis |  | |  |  |  |  |  |  |  |  |
| 26 | carditis |  | |  |  |  |  |  |  |  |  |
| 27 | endocarditis |  | |  |  |  |  |  |  |  |  |
| 28 | pleuropericarditis |  | |  |  |  |  |  |  |  |  |
| 29 | atypical mycobacterium pericarditis |  | |  |  |  |  |  |  |  |  |
| 30 | cytomegalovirus pericarditis |  | |  |  |  |  |  |  |  |  |
| 31 | endocarditis candida |  | |  |  |  |  |  |  |  |  |
| 32 | endocarditis histoplasma |  | |  |  |  |  |  |  |  |  |
| 33 | endocarditis q fever |  | |  |  |  |  |  |  |  |  |
| 34 | fungal endocarditis |  | |  |  |  |  |  |  |  |  |
| 35 | pericarditis fungal |  | |  |  |  |  |  |  |  |  |
| 36 | pericarditis histoplasma |  | |  |  |  |  |  |  |  |  |
| 37 | pericarditis tuberculous |  | |  |  |  |  |  |  |  |  |
| 38 | endocarditis enterococcal |  | |  |  |  |  |  |  |  |  |
| 39 | endocarditis haemophilus |  | |  |  |  |  |  |  |  |  |
| 40 | endocarditis pseudomonal |  | |  |  |  |  |  |  |  |  |
| 41 | endocarditis staphylococcal |  | |  |  |  |  |  |  |  |  |
| 42 | endocarditis viral |  | |  |  |  |  |  |  |  |  |
| 43 | streptococcal endocarditis |  | |  |  |  |  |  |  |  |  |
| 44 | viral pericarditis |  | |  |  |  |  |  |  |  |  |
| 45 | lupus endocarditis |  | |  |  |  |  |  |  |  |  |
| 46 | pericarditis lupus |  | |  |  |  |  |  |  |  |  |
| 47 | autoimmune pericarditis |  | |  |  |  |  |  |  |  |  |
| 48 | pericarditis malignant |  | |  |  |  |  |  |  |  |  |
| 49 | camptodactyly-arthropathy-coxa vara-pericarditis syndrome | | |  |  |  |  |  |  |  |  |
| 50 | encephalitis brain stem |  | |  |  |  |  |  |  |  |  |
| 51 | cerebral arteritis |  | |  |  |  |  |  |  |  |  |
| 52 | english: cardiomyopathy (smq) |  | |  |  |  |  |  |  |  |  |
| 53 | cardiomyopathy |  | |  |  |  |  |  |  |  |  |
| 54 | cardiomyopathy acute |  | |  |  |  |  |  |  |  |  |
| 55 | cardiomyopathy alcoholic |  | |  |  |  |  |  |  |  |  |
| 56 | cardiomyopathy neonatal |  | |  |  |  |  |  |  |  |  |
| 57 | chagas' cardiomyopathy |  | |  |  |  |  |  |  |  |  |
| 58 | congestive cardiomyopathy |  | |  |  |  |  |  |  |  |  |
| 59 | diabetic cardiomyopathy |  | |  |  |  |  |  |  |  |  |
| 60 | hiv cardiomyopathy |  | |  |  |  |  |  |  |  |  |
| 61 | hypertensive cardiomyopathy |  | |  |  |  |  |  |  |  |  |
| 62 | hypertrophic cardiomyopathy |  | |  |  |  |  |  |  |  |  |
| 63 | ischaemic cardiomyopathy |  | |  |  |  |  |  |  |  |  |
| 64 | metabolic cardiomyopathy |  | |  |  |  |  |  |  |  |  |
| 65 | non-obstructive cardiomyopathy |  | |  |  |  |  |  |  |  |  |
| 66 | obesity cardiomyopathy |  | |  |  |  |  |  |  |  |  |
| 67 | peripartum cardiomyopathy |  | |  |  |  |  |  |  |  |  |
| 68 | restrictive cardiomyopathy |  | |  |  |  |  |  |  |  |  |
| 69 | stress cardiomyopathy |  | |  |  |  |  |  |  |  |  |
| 70 | tachycardia induced cardiomyopathy |  | |  |  |  |  |  |  |  |  |
| 71 | thyrotoxic cardiomyopathy |  | |  |  |  |  |  |  |  |  |
| 72 | toxic cardiomyopathy |  | |  |  |  |  |  |  |  |  |
| 73 | viral cardiomyopathy |  | |  |  |  |  |  |  |  |  |
| 74 | i:\all pts\smq_sep/cardiomyopathy (smq).csv | | |  |  |  |  |  |  |  |  |
| 75 | camptodactyly-arthropathy-coxa vara-pericarditis syndrome | | |  |  |  |  |  |  |  |  |
| 76 | non-compaction cardiomyopathy |  | |  |  |  |  |  |  |  |  |
| 77 | cerebral amyloid angiopathy |  | |  |  |  |  |  |  |  |  |
| 78 | cerebral microangiopathy |  | |  |  |  |  |  |  |  |  |
| 79 | english: myocardial infarction (smq) |  | |  |  |  |  |  |  |  |  |
| 80 | acute myocardial infarction |  | |  |  |  |  |  |  |  |  |
| 81 | myocardial infarction |  | |  |  |  |  |  |  |  |  |
| 82 | post procedural myocardial infarction |  | |  |  |  |  |  |  |  |  |
| 83 | silent myocardial infarction |  | |  |  |  |  |  |  |  |  |
| 84 | periprocedural myocardial infarction |  | |  |  |  |  |  |  |  |  |
| 85 | ecg signs of myocardial infarction |  | |  |  |  |  |  |  |  |  |
| 86 | brain stem infarction |  | |  |  |  |  |  |  |  |  |
| 87 | ischaemic cerebral infarction |  | |  |  |  |  |  |  |  |  |
| 88 | cerebellar infarction |  | |  |  |  |  |  |  |  |  |
| 89 | cerebral infarction |  | |  |  |  |  |  |  |  |  |
| 90 | cerebral infarction foetal |  | |  |  |  |  |  |  |  |  |
| 91 | cerebral microinfarction |  | |  |  |  |  |  |  |  |  |
| 92 | embolic cerebellar infarction |  | |  |  |  |  |  |  |  |  |
| 93 | embolic cerebral infarction |  | |  |  |  |  |  |  |  |  |
| 94 | haemorrhagic cerebral infarction |  | |  |  |  |  |  |  |  |  |
| 95 | thrombotic cerebral infarction |  | |  |  |  |  |  |  |  |  |
| 96 | subendocardial haemorrhage |  | |  |  |  |  |  |  |  |  |
| 97 | myocardial haemorrhage |  | |  |  |  |  |  |  |  |  |
| 98 | pericardial haemorrhage |  | |  |  |  |  |  |  |  |  |
| 99 | brain stem haemorrhage |  | |  |  |  |  |  |  |  |  |
| 100 | brain stem microhaemorrhage |  | |  |  |  |  |  |  |  |  |
| 101 | english: central nervous system haemorrhages and cerebrovascular conditions (smq) | | | | |  |  |  |  |  |  |
| 102 | english: conditions associated with central nervous system haemorrhages and cerebrovascular accidents (smq) | | | | | |  |  |  |  |  |
| 103 | cerebellar haemorrhage |  | |  |  |  |  |  |  |  |  |
| 104 | cerebellar microhaemorrhage |  | |  |  |  |  |  |  |  |  |
| 105 | cerebral cyst haemorrhage |  | |  |  |  |  |  |  |  |  |
| 106 | cerebral haemorrhage |  | |  |  |  |  |  |  |  |  |
| 107 | cerebral haemorrhage foetal |  | |  |  |  |  |  |  |  |  |
| 108 | cerebral haemorrhage neonatal |  | |  |  |  |  |  |  |  |  |
| 109 | cerebral microhaemorrhage |  | |  |  |  |  |  |  |  |  |
| 110 | intraventricular haemorrhage |  | |  |  |  |  |  |  |  |  |
| 111 | intraventricular haemorrhage neonatal |  | |  |  |  |  |  |  |  |  |
| 112 | periventricular haemorrhage neonatal |  | |  |  |  |  |  |  |  |  |
| 113 | brain stem stroke |  | |  |  |  |  |  |  |  |  |
| 114 | cerebellar stroke |  | |  |  |  |  |  |  |  |  |
| 115 | myocardial fibrosis |  | |  |  |  |  |  |  |  |  |
| 116 | endocardial fibroelastosis |  | |  |  |  |  |  |  |  |  |
| 117 | cardiac fibrillation |  | |  |  |  |  |  |  |  |  |
| 118 | ventricular fibrillation |  | |  |  |  |  |  |  |  |  |
| 119 | cardiac arrest |  | |  |  |  |  |  |  |  |  |
| 120 | cardiac arrest neonatal |  | |  |  |  |  |  |  |  |  |
| 121 | cardio-respiratory arrest |  | |  |  |  |  |  |  |  |  |
| 122 | cardio-respiratory arrest neonatal |  | |  |  |  |  |  |  |  |  |
| 123 | post cardiac arrest syndrome |  | |  |  |  |  |  |  |  |  |
| 124 | foetal cardiac arrest |  | |  |  |  |  |  |  |  |  |
| 125 | english: cardiac failure (smq) |  | |  |  |  |  |  |  |  |  |
| 126 | cardiopulmonary failure |  | |  |  |  |  |  |  |  |  |
| 127 | cardiac failure |  | |  |  |  |  |  |  |  |  |
| 128 | cardiac failure acute |  | |  |  |  |  |  |  |  |  |
| 129 | cardiac failure chronic |  | |  |  |  |  |  |  |  |  |
| 130 | cardiac failure congestive |  | |  |  |  |  |  |  |  |  |
| 131 | cardiac failure high output |  | |  |  |  |  |  |  |  |  |
| 132 | neonatal cardiac failure |  | |  |  |  |  |  |  |  |  |
| 133 | radiation associated cardiac failure |  | |  |  |  |  |  |  |  |  |
| 134 | i:\all pts\smq_sep/cardiac failure (smq).csv |  | |  |  |  |  |  |  |  |  |
| 135 | cerebral circulatory failure |  | |  |  |  |  |  |  |  |  |
| 136 | acute left ventricular failure |  | |  |  |  |  |  |  |  |  |
| 137 | acute right ventricular failure |  | |  |  |  |  |  |  |  |  |
| 138 | chronic left ventricular failure |  | |  |  |  |  |  |  |  |  |
| 139 | chronic right ventricular failure |  | |  |  |  |  |  |  |  |  |
| 140 | left ventricular failure |  | |  |  |  |  |  |  |  |  |
| 141 | right ventricular failure |  | |  |  |  |  |  |  |  |  |
| 142 | ventricular failure |  | |  |  |  |  |  |  |  |  |
| 143 | prosthetic cardiac valve thrombosis |  | |  |  |  |  |  |  |  |  |
| 144 | congenital heart valve disorder |  | |  |  |  |  |  |  |  |  |
| 145 | congenital heart valve incompetence |  | |  |  |  |  |  |  |  |  |

**TT:** testosterone treatment; **MACE**: major adverse cardiovascular event; **N:** case number; **%**: percentage of case number. **Time to onset**: timespan since the start date of therapy to the event date
