# Supplementary material for: Major adverse cardiovascular events associated with testosterone treatment: a pharmacovigilance study of the FAERS database
Source: Front Pharmacol. 2023 Jul 12;14:1182113. doi: 10.3389/fphar.2023.1182113 (PMC10370495; doi:10.3389/fphar.2023.1182113)
Supplement: Supplementary file 2 [file Table2.docx]

**Supplement 2. Detail of bubble map：**IC_025_ generated by MACEs paired with various risk factors**.**

| Trim(pt) | TT | TT >50 yo | TT 18-49 yo | TT 0-17 yo | NT >50 yo | NT >65 yo | TD/Low-T | Anxiety | Cholesterol Increased | Depression | Diabetes | Gastrooesophageal Reflux | Hypertension | Hypothyroidism | Narcolepsy |
| --- | --- | --- | --- | --- | --- | --- | --- | --- | --- | --- | --- | --- | --- | --- | --- |
| Brain Stem Stroke | 0.30 | 0.00 | 0.00 | 0.00 | 0.54 | 0.22 | 0.27 | 0.00 | 0.00 | 0.00 | 1.65 | 0.00 | 0.00 | 0.00 | 0.00 |
| - |  |  |  |  |  |  |  |  |  |  |  |  |  |  |  |
| Cardiac Death | 1.77 | 0.00 | 2.58 | 0.00 | 0.61 | 0.87 | 0.00 | 0.00 | 0.00 | 0.00 | 1.67 | 0.00 | 0.00 | 0.00 | 0.00 |
| Sudden Cardiac Death | 1.95 | 0.98 | 0.88 | 0.00 | 0.70 | 0.61 | 1.48 | 0.07 | 0.00 | 0.00 | 2.35 | 0.00 | 0.74 | 0.00 | 0.00 |
| - |  |  |  |  |  |  |  |  |  |  |  |  |  |  |  |
| Cardiac Arrest | 0.88 | 0.31 | 0.00 | 0.00 | 0.69 | 0.68 | 0.59 | 0.00 | 0.00 | 0.00 | 0.83 | 0.00 | 0.62 | 0.17 | 0.00 |
| Ventricular Fibrillation | 1.22 | 0.55 | 0.00 | 0.00 | 0.90 | 0.82 | 1.00 | 0.00 | 1.29 | 0.52 | 1.09 | 0.00 | 0.73 | 0.62 | 0.95 |
| Acute Left Ventricular Failure | 0.66 | 0.00 | 0.00 | 0.00 | 0.53 | 0.72 | 0.00 | 0.00 | 0.00 | 0.00 | 2.00 | 0.00 | 1.03 | 0.00 | 0.00 |
| Left Ventricular Failure | 0.50 | 1.19 | 0.00 | 0.00 | 0.77 | 0.94 | 0.35 | 0.00 | 0.00 | 0.00 | 0.84 | 0.00 | 1.54 | 0.51 | 0.00 |
| Cardiac Failure | 0.05 | 0.00 | 0.00 | 0.00 | 0.83 | 1.20 | 0.00 | 0.00 | 0.00 | 0.00 | 1.07 | 0.00 | 1.36 | 0.89 | 0.00 |
| Cardiac Failure Acute | 0.92 | 0.00 | 0.79 | 0.00 | 0.92 | 1.22 | 0.00 | 0.00 | 0.00 | 0.00 | 1.12 | 0.00 | 1.66 | 0.26 | 0.00 |
| - |  |  |  |  |  |  |  |  |  |  |  |  |  |  |  |
| Cardiac Failure Congestive | 1.10 | 0.81 | 0.00 | 0.00 | 0.76 | 0.84 | 0.99 | 0.00 | 0.00 | 0.00 | 3.05 | 0.00 | 0.66 | 0.02 | 0.00 |
| Cardiomyopathy | 2.12 | 1.54 | 1.27 | 0.00 | 0.32 | 0.18 | 1.43 | 0.00 | 0.12 | 0.00 | 1.52 | 0.00 | 0.00 | 0.00 | 0.00 |
| Congestive Cardiomyopathy | 1.50 | 0.00 | 2.45 | 0.00 | 0.55 | 0.33 | 0.00 | 0.00 | 0.00 | 1.28 | 1.32 | 0.00 | 0.28 | 0.73 | 0.00 |
| Ischaemic Cardiomyopathy | 3.02 | 2.60 | 0.02 | 0.00 | 1.42 | 1.38 | 2.99 | 0.03 | 0.64 | 0.00 | 3.60 | 0.09 | 0.71 | 0.00 | 0.00 |
| Endocarditis | 0.28 | 0.00 | 0.92 | 0.00 | 0.70 | 0.70 | 0.00 | 0.00 | 0.00 | 0.00 | 0.00 | 0.00 | 0.76 | 0.00 | 0.00 |
| Myocardial Fibrosis | 1.13 | 0.00 | 2.72 | 0.00 | 0.24 | 0.00 | 0.00 | 0.00 | 0.00 | 0.00 | 0.00 | 0.00 | 0.00 | 0.00 | 0.00 |
| - |  |  |  |  |  |  |  |  |  |  |  |  |  |  |  |
| Acute Myocardial Infarction | 4.26 | 3.35 | 2.71 | 0.00 | 1.26 | 1.24 | 4.08 | 0.08 | 0.63 | 0.15 | 2.84 | 0.39 | 1.42 | 1.12 | 0.00 |
| Myocardial Infarction | 3.12 | 2.24 | 1.63 | 0.00 | 0.80 | 0.60 | 2.74 | 0.00 | 0.91 | 0.00 | 2.66 | 0.00 | 0.21 | 0.00 | 0.00 |
| Cerebellar Infarction | 2.18 | 1.31 | 0.33 | 0.00 | 0.89 | 0.96 | 1.90 | 0.00 | 0.00 | 0.00 | 1.41 | 0.00 | 0.76 | 0.00 | 0.00 |
| Cerebral Infarction | 1.43 | 0.84 | 0.13 | 0.00 | 1.02 | 1.33 | 1.17 | 0.00 | 0.00 | 0.00 | 1.49 | 0.00 | 1.48 | 0.44 | 0.00 |
| Ischaemic Cerebral Infarction | 1.38 | 0.78 | 0.00 | 0.00 | 0.56 | 0.51 | 1.40 | 0.00 | 0.00 | 0.02 | 0.00 | 0.00 | 0.61 | 0.00 | 0.00 |
| Brain Stem Infarction | 1.57 | 1.31 | 0.00 | 0.00 | 0.97 | 1.04 | 1.41 | 0.00 | 0.00 | 0.00 | 2.06 | 0.00 | 1.76 | 0.00 | 0.00 |
| - |  |  |  |  |  |  |  |  |  |  |  |  |  |  |  |
| Arterial Thrombosis | 0.25 | 0.00 | 0.00 | 0.00 | 0.52 | 0.42 | 0.00 | 0.00 | 0.00 | 0.00 | 0.00 | 0.00 | 0.00 | 0.00 | 0.00 |
| Cardiac Ventricular Thrombosis | 1.36 | 0.00 | 0.00 | 0.00 | 0.81 | 1.00 | 0.00 | 0.00 | 0.00 | 0.00 | 0.24 | 0.00 | 0.00 | 0.00 | 0.00 |
| Cerebral Thrombosis | 0.78 | 0.00 | 0.00 | 0.00 | 0.13 | 0.28 | 0.02 | 0.00 | 0.00 | 0.00 | 0.27 | 0.00 | 0.10 | 0.00 | 0.00 |
| Cerebral Venous Thrombosis | 1.15 | 0.66 | 0.00 | 0.00 | 0.00 | 0.00 | 0.00 | 0.00 | 0.00 | 0.00 | 0.00 | 0.00 | 0.00 | 0.00 | 0.00 |
| Coronary Artery Thrombosis | 2.92 | 1.89 | 1.38 | 0.00 | 0.96 | 0.60 | 1.68 | 0.00 | 0.34 | 0.00 | 0.10 | 0.00 | 0.12 | 0.00 | 0.00 |
| Coronary Bypass Thrombosis | 0.09 | 0.54 | 0.00 | 0.00 | 0.00 | 0.00 | 0.00 | 0.00 | 0.00 | 0.00 | 0.00 | 0.00 | 0.60 | 0.00 | 0.00 |
| Deep Vein Thrombosis | 4.05 | 3.11 | 2.73 | 0.00 | 0.12 | 0.09 | 3.76 | 0.00 | 0.00 | 0.00 | 0.00 | 0.00 | 0.00 | 0.00 | 0.00 |
| Thrombosis | 2.47 | 1.75 | 1.23 | 0.00 | 0.17 | 0.22 | 2.25 | 0.00 | 0.00 | 0.00 | 0.00 | 0.00 | 0.00 | 0.00 | 0.00 |

TT: testosterone treatment; NT: no record of TT was found; TD: testosterone deficiency; low-T: low testosterone level; yo: year-old
