# Supplementary material for: Major adverse cardiovascular events associated with testosterone treatment: a pharmacovigilance study of the FAERS database
Source: Front Pharmacol. 2023 Jul 12;14:1182113. doi: 10.3389/fphar.2023.1182113 (PMC10370495; doi:10.3389/fphar.2023.1182113)
Supplement: Supplementary file 3 [file Table3.docx]

**Supplement table 3.** Chi square test for IC_025_ generated by MACEs paired with various risk factors.

| p_val | TT | TD | TT >50 yo | TT 18-49 yo | NT >50 yo | NT >65 yo | Diabetes | Hypertension |
| --- | --- | --- | --- | --- | --- | --- | --- | --- |
| TT | - | 1.0000 | 1.0000 | 0.8786 | 0.9988 | 0.9909 | 0.9792 | 0.8937 |
| TD | 1.0000 | - | 1.0000 | 0.4000 | 0.9521 | 0.8793 | 0.9602 | 0.7670 |
| TT >50 yo | 1.0000 | 1.0000 | - | 0.5369 | 0.9698 | 0.9262 | 0.9463 | 0.9008 |
| TT 18-49 yo | 0.8786 | 0.4000 | 0.5369 | - | 0.7892 | 0.7429 | 0.6684 | 0.6649 |
| NT >50 yo | 0.9988 | 0.9521 | 0.9698 | 0.7892 | - | 1.0000 | 0.9997 | 1.0000 |
| NT >65 yo | 0.9909 | 0.8793 | 0.9262 | 0.7429 | 1.0000 | - | 0.9985 | 1.0000 |
| Diabetes | 0.9792 | 0.9602 | 0.9463 | 0.6684 | 0.9997 | 0.9985 | - | 0.9777 |
| Hypertension | 0.8937 | 0.7670 | 0.9008 | 0.6649 | 1.0000 | 1.0000 | 0.9777 | - |

TT: testosterone treatment; NT: no record of TT was found; TD: testosterone deficiency; low-T: low testosterone level; yo: year-old
