# Supplementary material for: Major adverse cardiovascular events associated with testosterone treatment: a pharmacovigilance study of the FAERS database
Source: Front Pharmacol. 2023 Jul 12;14:1182113. doi: 10.3389/fphar.2023.1182113 (PMC10370495; doi:10.3389/fphar.2023.1182113)
Supplement: Supplementary file 4 [file Table4.docx]

**Supplement table 4.** Trend of signal, take myocardial infarction as an example.

| **MI:** | 2004.00 | 2005.00 | 2006.00 | 2007.00 | 2008.00 | 2009.00 | 2010.00 | 2011.00 | 2012.00 | 2013.00 | 2014.00 | 2015.00 | 2016.00 | 2017.00 | 2018.00 | 2019.00 | 2020.00 | 2021.00 | 2022.00 |
| --- | --- | --- | --- | --- | --- | --- | --- | --- | --- | --- | --- | --- | --- | --- | --- | --- | --- | --- | --- |
|  |  |  |  |  |  |  |  |  |  |  |  |  |  |  |  |  |  |  |  |
| IC025 acm | -1.18 | -1.45 | -2.27 | -1.41 | -0.54 | -1.55 | -1.10 | -1.54 | -1.22 | -0.99 | 0.92 | 2.15 | 3.78 | 3.05 | 3.89 | 1.17 | 0.46 | -0.86 | 0.03 |
| **IC025 sep** | **-1.18** | **-1.16** | **-1.13** | **-0.99** | **-0.76** | **-0.80** | **-0.75** | **-0.74** | **-0.76** | **-0.73** | **0.27** | **1.17** | **1.71** | **1.84** | **1.97** | **1.99** | **2.00** | **2.01** | **2.04** |
|  |  |  |  |  |  |  |  |  |  |  |  |  |  |  |  |  |  |  |  |
| ln ROR acm | -0.13 | -0.53 | -0.96 | -0.26 | 0.35 | -0.49 | -0.33 | -0.59 | -0.44 | -0.26 | 0.80 | 1.64 | 2.83 | 2.36 | 2.91 | 1.16 | 0.72 | -0.02 | 0.54 |
| **ln ROR sep** | **-0.13** | **-0.41** | **-0.45** | **-0.37** | **-0.24** | **-0.30** | **-0.29** | **-0.31** | **-0.34** | **-0.34** | **0.30** | **0.89** | **1.26** | **1.35** | **1.44** | **1.46** | **1.46** | **1.47** | **1.48** |

IC_025_: lower limit of information component; ln ROR: [natural logarithm](javascript:;) value of ROR; acm: calculation based on dataset accumulated year after year; sep: calculation based on dataset of every single year.
